# Human Motion Segmentation via Spatiotemporally Dual-Constrained Density Estimation with Commodity Wi-Fi Device

**DOI:** 10.3390/s26113303

**Published:** 2026-05-22

**Authors:** Xu Wang, Linghua Zhang, Feng Shu

**Affiliations:** 1 School of Communications and Information Engineering, Nanjing University of Posts and Telecommunications, Nanjing 210003, China; wangxhhtc@163.com (X.W.); 2018010101@njupt.edu.cn (F.S.); 2 School of Physics, Electronics and Intelligent Manufacturing, Huaihua University, Huaihua 418000, China; 3 Jiangsu Engineering Research Center of Communication and Network Technology, Nanjing 210003, China

**Keywords:** channel state information, internet of things, motion segmentation, Wi-Fi

## Abstract

In ubiquitous Wi-Fi sensing, human motion interval segmentation is crucial for applications ranging from basic intrusion detection to advanced activity understanding. Existing methods often treat the Channel State Information (CSI) primarily as time series, overlooking its rich information in the spatial and frequency domains. To address this, we propose a training-free motion segmentation method that exploits the spatiotemporal features of CSI. We first analyze the discriminative spatial distributions of the CSI Ratio on the complex plane and construct a spatiotemporally dual-constrained local density estimator to characterize motion-induced perturbations. To overcome subcarrier selection challenges, we introduce a packet-level asymmetric truncation-based fusion algorithm, which yields a feature representation with a pronounced bimodal histogram. This enables the automatic determination of the optimal segmentation threshold based on the distribution characteristics of the truncated density image. Experiments in typical indoor environments demonstrate that the proposed method achieves high accuracy in both motion event detection and interval localization.

## 1. Introduction

Advancements in human-centric and pervasive computing increasingly rely on accurate and robust environment sensing. Compared to traditional vision-based or wearable solutions [1,2], device-free sensing using ubiquitous Wi-Fi signals holds significant advantages in terms of non-intrusiveness, low cost, and privacy preservation. Among various Wi-Fi sensing tasks, motion interval segmentation is a fundamental component, aiming to accurately extract the time segments of human activities from continuous wireless signals. Reliably distinguishing between static and dynamic states is not only a prerequisite for basic applications including intrusion detection and area surveillance [3,4] but also a key enabler for advanced fine-grained capabilities such as activity recognition and indoor localization [5,6,7,8].

Human movement alters the wireless propagation channel. Consequently, research commonly uses the Received Signal Strength Indicator (RSSI) or Channel State Information (CSI) to sense human motion. Compared with RSSI, which conveys only coarse-grained power measurements, CSI offers amplitude and phase information at the subcarrier level, giving it finer-grained sensing potential. To quantify channel fluctuations caused by human motion, existing methods typically compute the variance or correlation of the CSI series [9]. However, two key challenges persist in these methods. First, due to frequency-selective fading, different subcarriers exhibit varying sensitivity to environmental changes, making effective subcarrier selection critical to system performance. Second, training-free threshold determination remains difficult since the environmental noise changes over time or across scenarios. Moreover, unlike images or text, CSI is time-series data that is costly to acquire and highly environment specific [10]. This makes constructing large-scale, diverse labeled datasets for real-world scenarios both time-consuming and labor-intensive, thereby limiting the practicality of data-hungry deep learning methods.

To address these challenges, this paper presents a training-free human motion segmentation framework that leverages both the spatial and temporal characteristics of CSI. By re-analyzing the distribution of CSI Ratio sample points on the complex plane, this work first develops an improved KNN density estimation with additional temporal constraints. Then, a packet-level asymmetric truncation mechanism is employed to eliminate subcarriers with poor static stability and dynamic sensitivity. This yields a feature representation with a distinct bimodal histogram, upon which motion intervals are segmented using the efficient Valley-Emphasized Otsu (VE-Otsu) algorithm. In contrast to our prior works [11,12], the proposed method not only achieves complete and accurate motion interval segmentation in a single pass but also requires neither additional static data collection nor complex iterative procedures.

The main contributions of this work are threefold:

- We propose a spatiotemporally constrained KNN density estimator to capture motion-induced CSI perturbations. Compared to the purely spatial constraints in the original KNN density estimation, the introduced temporal constraint avoids misleading neighbors from temporally distant samples, leading to a more distinct discrimination between static and dynamic states.
- We present a packet-level asymmetric truncation-based subcarrier fusion strategy to mitigate subcarrier dependence. Unlike commonly adopted subcarrier-level selection, the proposed packet-level fusion method selects subcarrier components with superior environmental perception at a finer granularity, which provides a critical foundation for training-free threshold calculation.
- We conduct systematic experiments to validate the effectiveness of the proposed method. Results from real-indoor scenarios demonstrate that our approach achieves both high motion event detection and interval localization accuracy, while requiring no model training.

The remainder of this paper is organized as follows. Section 2 reviews related works on Wi-Fi CSI-based human motion sensing. Section 3 introduces the background, including CSI and the derived CSI Ratio. Section 4 details the core methodology and the complete processing pipeline. Section 5 presents a comprehensive experimental evaluation to validate the proposed approach. Finally, Section 6 concludes the paper and outlines potential future work.

## 2. Related Works

Fine-grained CSI has facilitated the development of high-precision motion sensing using ubiquitous Wi-Fi. Consequently, a variety of detection and segmentation methods have emerged, which are broadly classified into three categories: variance-based, correlation-based, and learning-based approaches.

**Variance-based Methods.** Variance-based human motion detection exploits the difference in CSI temporal fluctuation to discriminate between static and dynamic states. A series of works utilize the CSI variance in static states as a detection threshold, identifying segments where the variance exceeds this baseline as motion intervals [13,14,15]. Meanwhile, various improvements have been proposed for this basic framework, particularly regarding subcarrier selection and threshold adaptation. For example, MoSeFi [11] introduces a subcarrier selection criterion based on variance and curvature distance to support segmentation. Furthermore, it exploits the conformality of the Möbius transformation and defines a novel motion indicator from the real and imaginary parts of the CSI Ratio, which reduces motion fragmentation and enhances segmentation accuracy. Work [16] sets the initial threshold to the static variance obtained during system initialization and employs eigenvalue density estimation to build a static profile, mitigating threshold drift due to environmental changes. LightSeg [17] detects the end of human activity first using a coarse variance threshold, then decides the appropriate threshold based on the linear traversal of current CSI stream. WiSTD [18] computes a weighted average of the filtered CSI amplitude and detects motion by comparing the variance within a sliding window against a preset threshold. Since variance is inherently influenced by the absolute signal energy, these approaches typically require pre-collected static data to set an initial threshold, which limits their environmental adaptability.

**Correlation-based methods.** To enhance environmental adaptability, researchers have attempted to construct more robust motion indicators from the perspective of correlation, including the statistical relationships of CSI across time and frequency domains. The use of covariance represents a prominent approach. PADS [19] defines an environment-independent metric that approaches one in static states and deviates during motion. However, its limited discriminative power necessitates a Support Vector Machine (SVM) method for threshold determination. WiSH [20,21] forms a composite motion indicator by integrating temporal and frequency correlations and uses the static value as an adaptive threshold, enabling deployment on resource-constrained nodes without training. The Autocorrelation Function (ACF) represents another distinct approach for motion sensing. Wi-MoID [22] builds a motion detection statistic from the ACF of CSI amplitude but suffers from limited discrimination between static and dynamic states. To address this, SrcSense [23] and follow-up work [24] employ maximum ratio combining to compute an enhanced A-ACF and apply an empirical threshold for segmentation. Work [4] further trains a neural network on ACF features to recognize human presence patterns and use a sliding-window median filter to reduce false alarms. Separately, WiPIHT [25] utilizes the ACF to estimate real-time walking speed within an indoor localization framework so that the motion intervals can be segmented based on a predefined speed threshold. While correlation-based methods reduce environmental dependence, the robustness and universality of the segmentation threshold remain a persistent issue.

**Learning-based Methods.** The success of machine learning, especially deep learning, in fields like image and speech processing has motivated its adoption for Wi-Fi sensing. Some works [26,27,28] extract a set of handcrafted statistical measures from CSI (e.g., mean amplitude, variance, mean absolute deviation, range, interquartile range, coefficient of variation, and polar deviation), and employ traditional machine learning models like ensemble learning for motion detection. To circumvent the potential bias of manually designed features, researchers have leveraged neural networks to learn more discriminative representations. For example, DeepSeg [29] segments continuous CSI streams into fixed-length bins and employs a Convolutional Neural Network (CNN) to classify four human motion states. Work [30] computes the element-wise logarithm of a 2D Fourier transform and uses a deep clustering network with an embedded autoencoder for motion detection. LiteWiSys [31] employs an end-to-end deep learning framework that utilizes a multi-scale convolutional backbone for Wi-Fi-based human motion detection. Work [32] applies a self-organizing neural network to extract CSI features, followed by a Softmax classifier for state probability estimation. Furthermore, work [33] compares the motion detection performance of CNN, Long Short-Term Memory (LSTM) and CNN-LSTM models on CSI amplitude and temporal spectrum features. Typically, learning-based methods can achieve superior system performance. However, this comes at the cost of heavy data collection and annotation burdens due to their reliance on large-scale training data.

**Other Methods.** Meanwhile, several studies have explored motion detection and segmentation from alternative technical perspectives. Wi-Senser [34] detects large-scale body motions during sleep by identifying peaks in CSI amplitude fluctuations. WiDFS2.0 [35] applies a Doppler Fast Fourier Transform (FFT) along the time dimension to estimate frequency shifts and employs a Constant False Alarm Rate (CFAR) detector to identify moving targets. Work [36] leverages the dispersion of the CSI Ratio on the complex plane to detect human motion. Work [12] introduces a quasi-envelope feature for capturing CSI fluctuations and proposes an iterative segmentation method using an enhanced Otsu threshold. It further incorporates a motion detection algorithm to decide when to terminate the iteration. Work [37] constructs a motion feature by combining the Short Time Fourier Transform (STFT) and the Breath-to-Noise Ratio (BNR) for human detection. These works propose innovative solutions that collectively advance Wi-Fi sensing in specialized scenarios.

## 3. Background

### 3.1. CSI Fundamentals

In a typical indoor environment, the received Radio Frequency (RF) signal is a superposition of multipath components, i.e., one Line-of-Sight (LOS) path and multiple reflection paths. CSI characterizes the multipath propagation of the RF signal, which can be expressed as

(1) $$h\left(f,t\right)=\sum_{p=1}^{P}\alpha_{p}\exp\;\left(-j2\pi f\tau_{p}\left(t\right)\right)$$

where *f* is the frequency of the RF signal, $\alpha_{p}$ and $\tau_{p}\left(t\right)$ are the complex-valued attenuation coefficient and propagation delay of the $p_{th}$ path, respectively, and *P* is the number of paths.

According to prior work [38], the propagation paths can be divided into two parts: the static component, which includes the LOS path and multiple reflection paths from fixed objects, and the dynamic component, which refers to the reflection paths whose lengths change with the movement of the target. Thus, the CSI can be represented as

(2) $$\begin{array}{cc} h(f,t) & =h^{\left(s\right)}\left(f,t\right)+h^{\left(d\right)}\left(f,t\right)\\ & =\sum_{m=1}^{P^{\left(s\right)}}\alpha_{m}\exp\;\left(-j2\pi f\tau_{m}\left(t\right)\right)+\sum_{n=1}^{P^{\left(d\right)}}\alpha_{n}\exp\;\left(-j2\pi f\tau_{n}\left(t\right)\right) \end{array}$$

where the superscripts ^(*s*)^ and ^(*d*)^ denote the static and dynamic components, respectively. Under the condition of a single, regularly moving object, the dynamic component can be approximated as arising from a single dynamic reflection path [39]. Given that τ(t)=L(t)/fλ, where L(t) is the propagation path length and λ is the wavelength of the RF signal, Equation (2) can be simplified to

(3) $$h\left(f,t\right)=h^{\left(s\right)}\left(f,t\right)+\alpha^{\left(d\right)}\exp\;\left(-j2\pi L^{\left(d\right)}\left(t\right)/\lambda \right)$$

where $\alpha^{\left(d\right)}$ is the complex-valued attenuation coefficient of the dynamic reflection path $L^{\left(d\right)}\left(t\right)$. Since the static component $h^{\left(s\right)}\left(f,t\right)$ in the environment remains relatively constant, variations in reflection path length caused by moving objects directly induce fluctuations in the CSI. This makes CSI an effective tool for passive environmental sensing.

In practical Wi-Fi systems, the acquired CSI is predominantly corrupted by two types of hardware noise: amplitude noise caused by Automatic Gain Control (AGC) fluctuations, and phase noise arising from the clock asynchrony between the transmitter and receiver, including Carrier Frequency Offset (CFO), Sampling Frequency Offset (SFO), and Symbol Timing Offset (STO). Therefore, the received CSI can be modeled as

(4) $$\begin{array}{cc} \tilde{h}\left(f,t\right) & =\Delta_{AGC}\cdot \exp\left(-j2\pi \theta_{noise}\right)\cdot h\left(f,t\right)\\ & =\Delta_{AGC}\cdot \exp\left(-j2\pi \theta_{noise}\right)\cdot \left(h^{\left(s\right)}\left(f,t\right)+\alpha^{\left(d\right)}\exp\;\left(-j2\pi L^{\left(d\right)}\left(t\right)/\lambda \right)\right) \end{array}$$

where $\Delta_{AGC}$ represents the amplitude noise due to AGC variations, and $\theta_{noise}$ denotes the combined phase noise caused by CFO, SFO and STO. Since the CSI phase information $∠\tilde{h}\left(f,t\right)$ is severely corrupted by random noise, the CSI amplitude $\left|\tilde{h}\left(f,t\right)\right|$ becomes the primary usable component in practice.

### 3.2. From CSI to CSI Ratio

For a Multiple Input Multiple Output (MIMO) and Orthogonal Frequency Division Multiplexing (OFDM) Wi-Fi system, the CSI Ratio is defined as the quotient of CSI between two antennas [39]. Mathematically, it is

(5) $$\tilde{CR}\left(f,t\right)=\frac{{\tilde{h}}_{1}\left(f,t\right)}{{\tilde{h}}_{2}\left(f,t\right)}$$

where subscripts _1_ and _2_ are used to differentiate the collected CSI from antenna 1 and antenna 2, respectively.

According to prior works [40,41], the amplitude and phase noise between different antennas are correlated on a WiFi card because they share the same AGC and oscillator. Thus, Equation (5) simplifies to

(6) $$\begin{array}{cc} \tilde{CR}\left(f,t\right) & =\frac{\Delta_{AGC,1}\cdot \exp\left(-j2\pi \theta_{noise,1}\right)\cdot \left(h_{1}^{\left(s\right)}\left(f,t\right)+\alpha_{1}^{\left(d\right)}\exp\;\left(-j2\pi L_{1}^{\left(d\right)}\left(t\right)/\lambda \right)\right)}{\Delta_{AGC,2}\cdot \exp\left(-j2\pi \theta_{noise,2}\right)\cdot \left(h_{2}^{\left(s\right)}\left(f,t\right)+\alpha_{2}^{\left(d\right)}\exp\;\left(-j2\pi L_{2}^{\left(d\right)}\left(t\right)/\lambda \right)\right)}\\ \; & =\frac{h_{1}^{\left(s\right)}\left(f,t\right)+\alpha_{1}^{\left(d\right)}\exp\;\left(-j2\pi L_{1}^{\left(d\right)}\left(t\right)/\lambda \right)}{h_{2}^{\left(s\right)}\left(f,t\right)+\alpha_{2}^{\left(d\right)}\exp\;\left(-j2\pi L_{2}^{\left(d\right)}\left(t\right)/\lambda \right)}\\ & =\frac{h_{1}^{\left(s\right)}\left(f,t\right)+\alpha_{1}^{\left(d\right)}\exp\;\left(-j2\pi L_{1}^{\left(d\right)}\left(t\right)/\lambda \right)}{h_{2}^{\left(s\right)}\left(f,t\right)+\alpha_{2}^{\left(d\right)}\exp\;\left(-j2\pi \Delta L/\lambda \right)\exp\;\left(-j2\pi L_{1}^{\left(d\right)}\left(t\right)/\lambda \right)} \end{array}$$

where $\Delta L=L_{2}^{\left(d\right)}\left(t\right)-L_{1}^{\left(d\right)}\left(t\right)$ denotes the dynamic path length difference between the two antennas.

It can be seen that the complex-valued CSI Ratio, obtained through a simple division operation, automatically cancels the hardware-induced amplitude and phase noise, enabling the use of both its amplitude and phase for environmental sensing. For two close-by antennas, ΔL can be considered as a constant when the target moves a short distance [39]. Consequently, the CSI Ratio can be interpreted as a Möbius transformation. When the dynamic path length $L_{1}^{\left(d\right)}\left(t\right)$ varies by one wavelength, the trajectory of the CSI Ratio forms a circle on the complex plane.

### 3.3. ChallengeS of Training-Free Motion Interval Segmentation

Due to the high time and labor costs of collecting and annotating CSI data, developing training-free motion detection and segmentation methods is crucial for reducing the deployment costs of sensing systems. This goal hinges on two key aspects: constructing feature descriptors that effectively discriminate between static and dynamic states, and designing accurate, adaptive methods for calculating the segmentation threshold.

**Figure 1:**
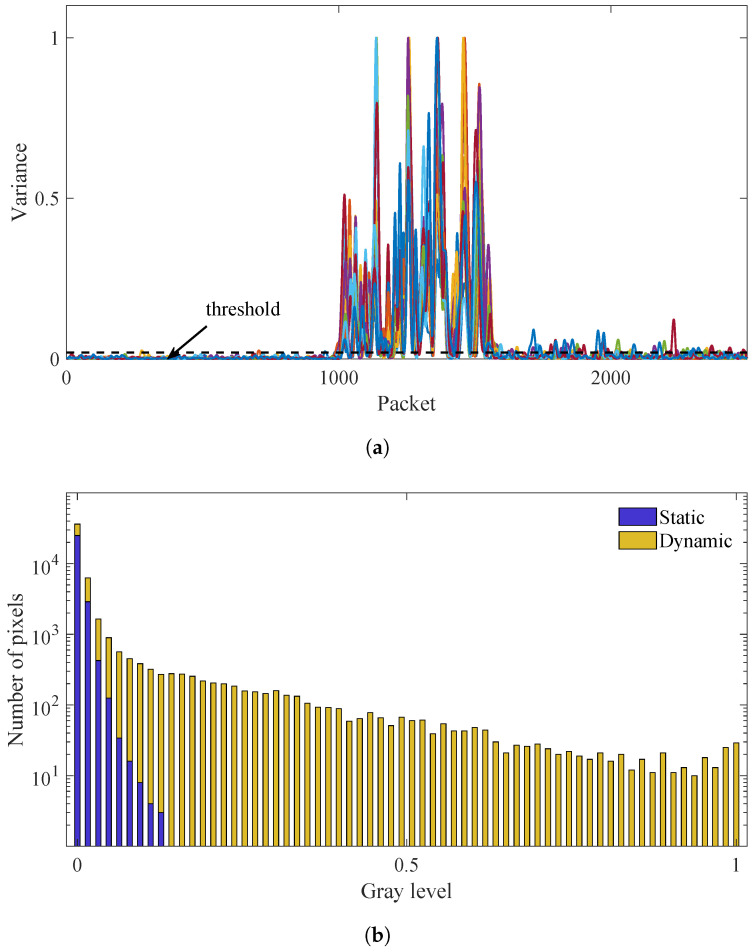
Measurement of CSI Ratio fluctuation. (**a**) Moving variance. (**b**) Histogram of moving variance.

To detect and segment time intervals containing moving objects, statistics such as variance or correlation coefficients are commonly used to quantify the fluctuation of CSI or CSI Ratio. Figure 1a shows the moving variance of the amplitude series. It can be seen that most values of static interval are larger than those of dynamic interval. Therefore, many existing methods—including our prior work MoSeFi [11]—determine the segmentation threshold using pre-collected static data. However, their performance is highly sensitive to the specific noise level present in that static reference data. As environmental noise varies over time or across scenarios, this empirically set threshold fails to generalize, which requires recurrent data collection and threshold recalibration.

On the other hand, human motion induces sinusoidal fluctuations in the amplitude or phase of the CSI Ratio series, resulting in an overlap between the variance distributions of static and dynamic intervals. As shown in Figure 1b, the variance values of static intervals are highly concentrated within a narrow range near zero, whereas those of motion intervals are distributed across a much wider range. In our prior work [12], deriving the envelope significantly improved the consistency within dynamic intervals and mitigated the overlap in the gray values. However, the envelope transform cannot fundamentally change the distribution ranges of the gray values for different states. For static intervals, the original variance series and its envelope are inherently very close. This skewed distribution increases the difficulty of segmenting motion intervals from an image perspective. Consequently, the method in [12] resorted to a complex iterative segmentation algorithm to obtain complete motion intervals.

## 4. Method

### 4.1. Motivation

**Figure 2:**
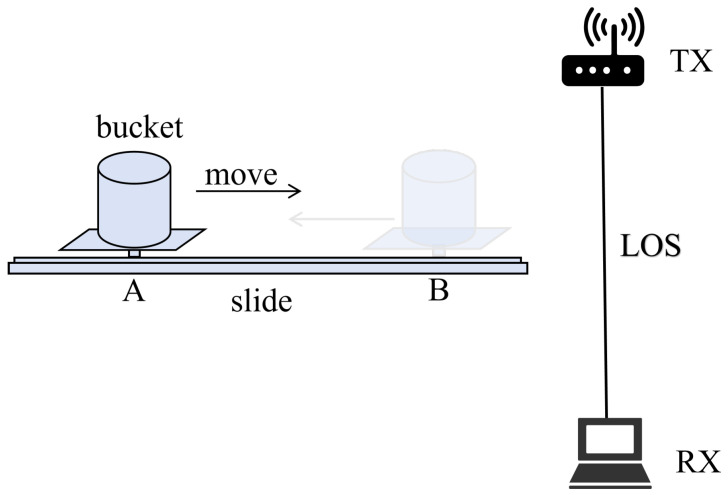
Schematic of the verification experiment.

A verification experiment was conducted to investigate the discriminatory features within the CSI Ratio. As shown in Figure 2, the transmitter and receiver were placed 1.5 m apart, with a slide positioned perpendicular to the LOS path. A metal bucket, controlled by a stepper motor, moved uniformly from point *A* to point *B* and then returned to point *A*.

**Figure 3:**
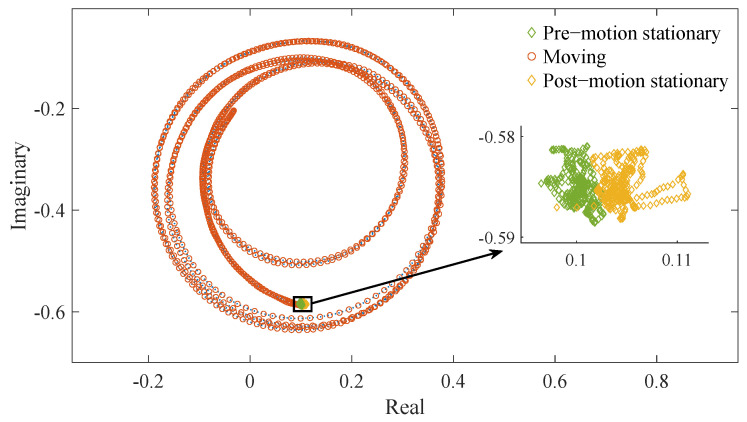
CSI Ratio distribution on the complex plane: bucket stationary vs. moving.

Figure 3 shows the trajectory of CSI Ratio sample points on the complex plane. When the metal bucket is stationary, no dynamic path is present, leaving only the static component in Equation (2). Under limited noise, the CSI Ratio sample points are localized around a single point on the complex plane as seen more clearly in the magnified view. In contrast, when the metal bucket moves, the CSI Ratio sample points trace a circle, which is a direct manifestation of the conformal property of the Möbius transformation. This is consistent with the description in Section 3.2.

On the other hand, the movement of a bucket induces distinctly different spatial distribution characteristics of the CSI Ratio on the complex plane. Specifically, the CSI Ratio sample points are densely distributed under static conditions, with a large number of neighbors in any finite neighborhood, whereas they become sparse under dynamic conditions, experiencing a sharp drop in the number of neighbors. Previous works primarily focus on analyzing the temporal stability of CSI amplitude and phase to detect and segment motion intervals. while these methods emphasize the fluctuation characteristics of the signal in the time domain but do not fully exploit the structural information inherent in the CSI Ratio within the complex plane. Therefore, analyzing the spatial distribution of the CSI Ratio emerges as a viable approach for understanding and segmenting motion intervals.

### 4.2. Perturbations Characterized via Density Estimation

Density estimation is a data-driven, nonparametric tool widely used in tasks like anomaly detection. Given its capability to reveal data distributions without assuming a predefined functional form, we attempt to leverage local density as a discriminative feature to characterize the different spatial distributions of the CSI Ratio on the complex plane. Common nonparametric density estimation methods include histogram-based, kernel-based and K-Nearest Neighbor (KNN)-based approaches [42]. In comparison, the KNN-based method adapts flexibly to any underlying Probability Density Function (PDF). Moreover, its parameter tuning is simple, as the only parameter to adjust is *K*.

Given a dataset *D* of *N* points, the K-distance $d_{K}\left(z_{i}\right)$ for a sample point $z_{i}\in D$ is defined as the distance between $z_{i}$ and its $K_{th}$ nearest neighbor. Correspondingly, the K-distance neighborhood can be defined as the set of all data points whose distance to $z_{i}$ is not greater than $d_{K}\left(z_{i}\right)$, i.e.,

(7) $$D_{neighbor}\left(z_{i}\right)=\left\{z\in D|\mathrm{dist}\left(z,z_{i}\right)\leq d_{K}\left(z_{i}\right)\right\}$$

where dist$\left(z_{1},z_{2}\right)$ is the distance metric between two points $z_{1}$ and $z_{2}$. Then, the KNN density estimator can be constructed as follows:

(8) $$den\left(z_{i}\right)=\frac{K}{NV(B\left(z_{i},d_{K}\left(z_{i}\right)\right))}$$

where $B(z_{i},d_{K}\left(z_{i}\right))$ denotes the ball centered at $z_{i}$ and radius $d_{K}\left(z_{i}\right)$, and $V(B\left(z_{i},d_{K}\left(z_{i}\right)\right))$ denotes the volume of $B(z_{i},d_{K}\left(z_{i}\right))$.

**Figure 4:**
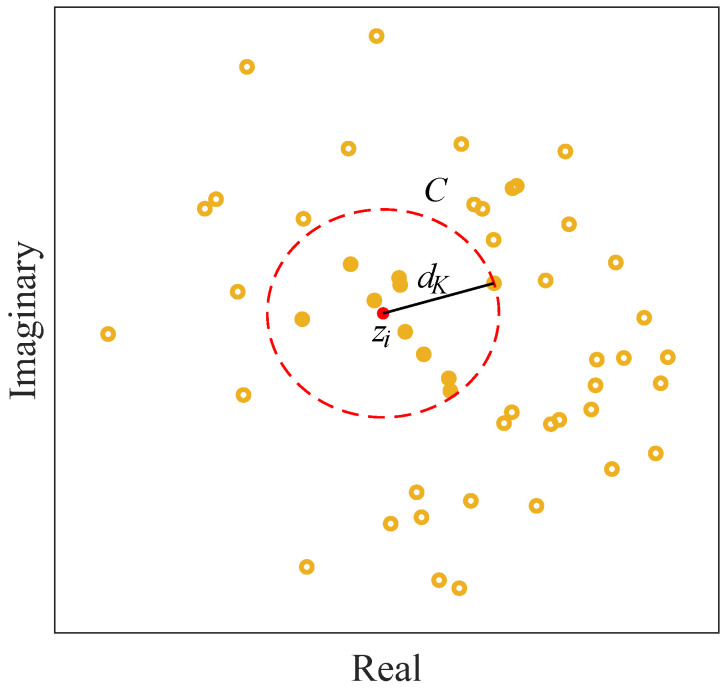
Schematic diagram of KNN density calculation.

As shown in Figure 4, for CSI Ratio sample points on the 2D complex plane, the K-distance neighborhood becomes a circular region. Thus, Equation (8) can be simplified to

(9) $$den\left(z_{i}\right)=\frac{K}{N\pi d_{K}^{2}\left(z_{i}\right)}$$

where the term $\pi d_{K}^{2}\left(z_{i}\right)$ represents the area of the K-distance neighborhood, which is the circle region *C* centered at $z_{i}$ with radius $d_{K}\left(z_{i}\right)$. The KNN density is inversely proportional to the area of *C*. For a fixed *K*, a denser sample point distribution around point $z_{i}$ yields a smaller $d_{K}\left(z_{i}\right)$, a smaller area *C*, and thus a higher density value. Conversely, a sparser distribution yields a lower density value.

**Figure 5:**
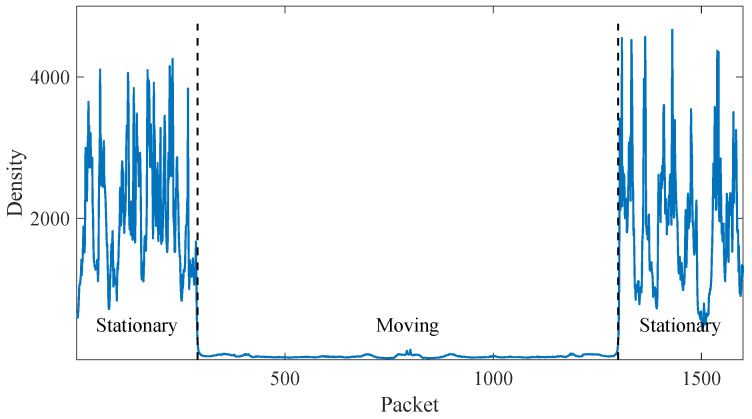
KNN density calculated for bucket moving and stationary.

Figure 5 shows the calculated KNN density series for the moving bucket experiment. It can be observed that the KNN density values computed for static intervals are significantly higher than those for dynamic intervals. This accurately characterizes the CSI Ratio distribution, which is dense during static periods but sparse during dynamic periods. Furthermore, unlike variance, whose values overlap in dynamic and static intervals, KNN density values during dynamic periods exhibit strong clustering. This provides a solid foundation for segmenting motion intervals. Therefore, by leveraging the spatial distribution of the CSI Ratio and quantifying it via KNN density, we obtain a promising feature for separating static and dynamic intervals.

### 4.3. Issues Posed by Human Motion Complexity

**Figure 6:**
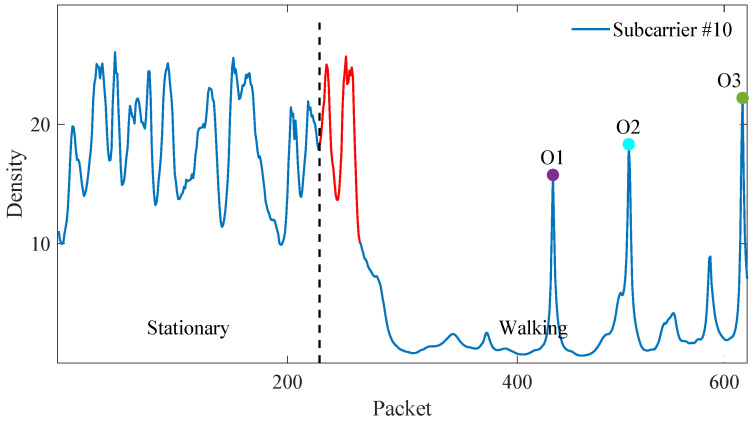
KNN density calculated for human walking and stationary.

To further assess the efficacy of density estimation, we compute the KNN density series for the human walking case, which is presented in Figure 6. However, the features of the KNN density series in this scenario differ from those observed in the moving bucket case. While the KNN density values are generally high when the human is stationary, they fail to remain consistently low during human walking. Similar to the case of variance, some values within motion intervals fall into the static distribution as demonstrated by the three marked points. This inevitably causes a fragmentation problem in threshold-based method. Moreover, during transitions between walking and stationary states, the KNN density is significantly overestimated as highlighted in red. Such overestimates cause inaccuracies in locating motion interval boundaries and, in severe cases, can even lead to the mis-segmentation of entire motion intervals.

**Figure 7:**
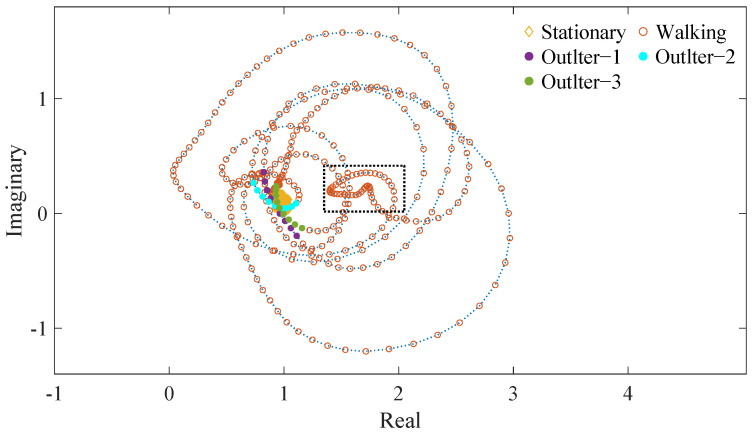
CSI Ratio distribution on the complex plane: human stationary vs. walking.

Unlike the simple, translational moving of the metal bucket—which can be regarded as a single-target displacement—human motion is far more complex. Taking walking as an example, it involves limb swings and torso movement. The concurrent movement of multiple parts of the human body disrupts the regular trajectory of the CSI Ratio. Figure 7 depicts the CSI Ratio distribution for the walking case. When the subject is stationary, the samples exhibit strong clustering and thus produce larger KNN density estimates. In contrast, when the human body is in motion, the trajectory of CSI Ratio samples deviates from a circular shape. This leads to two pronounced issues. First, several anomalously dense regions emerge within motion intervals as marked by the black box. Second, some sample points from motion intervals fall within the distribution range of static sample points as indicated by the colored markers. These issues directly cause an overestimation of density within motion intervals.

**Figure 8:**
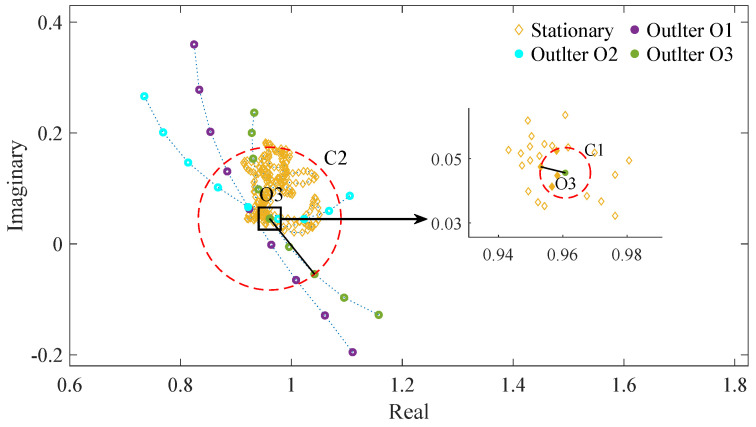
Schematic of KNN density calculation for outliers in motion intervals.

Figure 8 further illustrates the erroneous KNN density calculation within the motion interval. Taking the green outlier as an example, the trajectory disorder makes its $K_{th}$ nearest neighbor incorrectly fall within the distribution range of static sample points as shown by the yellow solid diamond in the enlarged view. Compared with the correct $K_{th}$ nearest neighbor located in the dynamic interval, which is shown by the green solid point, the area of erroneously K-distance neighborhood $C_{1}$ is significantly smaller than the right one $C_{2}$, thereby leading to a severely deviated KNN density estimate. Due to the inherent spatial proximity of boundary points to the static cluster, the same issue also affects the boundary region between static and dynamic states. While effective in simple scenarios, conventional KNN density estimation proves unreliable for distinguishing static and dynamic states in complex cases such as human motion.

### 4.4. Improved KNN Density Estimation with Spatiotemporal Constraints

Given that CSI is intrinsically a time series, the traditional KNN density estimator, which considers only spatial relationships while ignoring the associated temporal information, is evidently incomplete and unstable. To address this, we propose an improved KNN density estimation method incorporating spatiotemporal dual constraints.

Specifically, for the entire CSI Ratio sample set *D*, we define a subset under the packet index constraint, which can be expressed as

(10) $$D_{index}\left(z_{i}\right)=\left\{z\in D|index-diff\left(z,z_{i}\right)\leq N_{index}\right\}$$

where the operator index−diff denotes the packet index difference between two points in the CSI Ratio series, and $N_{index}$ is a predefined index difference threshold. Accordingly, the K-distance is redefined as the distance between $z_{i}$ and its $K_{th}$ nearest neighbor within the temporally constrained subset $D_{index}\left(z_{i}\right)$, rather than in *D*. The KNN density is then calculated using Equation (9).

**Figure 9:**
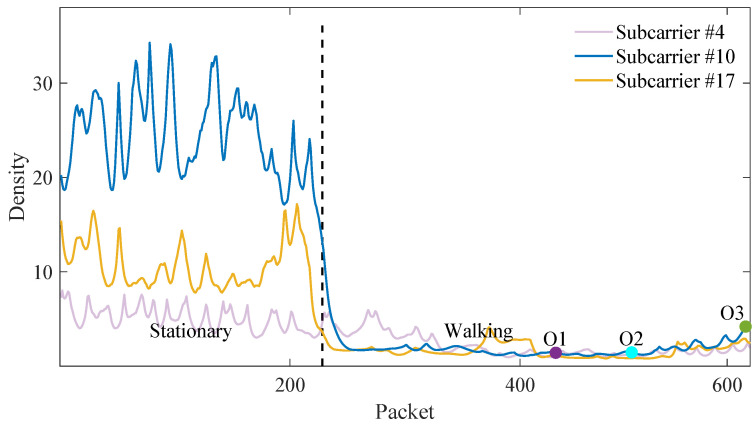
Local density estimation under spatiotemporal dual constraints.

The definition of $D_{index}\left(z_{i}\right)$ introduces temporal constraints to the KNN density calculation, thereby excluding the temporally distant sample points. Taking the outlier $O_{3}$ in Figure 8 as an example, because the packet-index difference between $O_{3}$ and the static cluster exceeds the threshold $N_{index}$, the $K_{th}$ nearest neighbor is selected from the dynamic interval (green hollow circle), even though static sample points are spatially closer. This results in the correct K-distance neighborhood $C_{2}$, thereby yielding an accurate local density estimate. Figure 9 shows the improved KNN density series computed with dual spatiotemporal constraints for the walking case. Compared with Figure 6, the introduction of temporal constraints effectively corrects the previous outliers in motion intervals and at transition boundaries, resulting in more consistent density estimates within the motion interval.

In the improved spatiotemporally constrained KNN density estimation, *K* and $N_{index}$ are two critical hyperparameters, where $K\leq 2N_{index}$. As indicated by Equation (9), the value of *K* directly governs the density calculation, but its impact is different in static and dynamic states. In the static state where CSI Ratio sample points are highly clustered, a larger *K* yields a significantly higher density value. This occurs because $d_{K}\left(z_{i}\right)$ increases only slowly with *K*, causing Equation (9) to be dominated by its numerator *K*. In contrast, within the dynamic state where sample points are sparse, increasing *K* leads to a reduction in the density estimate. The underlying reason is that the $d_{K}\left(z_{i}\right)$ grows substantially with *K*, making $d_{K}^{2}\left(z_{i}\right)$ the dominant term in Equation (9). Therefore, appropriately increasing *K* can enhance separability by amplifying the density contrast. However, *K* must remain below the number of static sample points. Otherwise, the density values in static states become indistinguishable from those in dynamic states.

On the other hand, the parameter $N_{index}$ controls the strength of the temporal constraints, which is the maximum temporal separation allowed when searching for the $K_{th}$ nearest neighbor. A larger $N_{index}$ weakens this constraint and increases the risk of outliers in dynamic states because it admits sample points from distorted trajectory segments of the CSI Ratio. While a smaller $N_{index}$ reduces outliers, it also imposes a correspondingly small upper bound on *K*, which compromises its ability to enhance discrimination between static and dynamic states. We employ a grid search to evaluate the impact of *K* and $N_{index}$ on segmentation performance, and a detailed analysis is provided in Section 5.5.1.

Moreover, the temporal constraint significantly reduces computational complexity. The original KNN density calculation requires computing pairwise distances over the full set *D* of size *N*, incurring $O(N^{2})$ time and space complexity. With the temporal constraint, the K-distance calculation is confined to the subset $D_{index}$ of size $N_{index}$, reducing time complexity to $O(NN_{index})$ and space complexity to O(N). Since $N_{index}\ll N$,the algorithm’s efficiency is substantially improved.

### 4.5. Packet-Level Asymmetric Truncation-Based Subcarrier Fusion

In MIMO-OFDM Wi-Fi systems, frequency-selective fading causes the environmental sensing capability to vary across subcarriers. As Figure 9 shows, subcarrier #10 exhibits superior static stability and dynamic sensitivity compared with the other two subcarriers. Identifying such an optimal subcarrier from numerous candidates is therefore critical for motion segmentation. Moreover, the density series is not directly usable for segmentation because the distribution ranges of the density series differ significantly due to the variations in noise levels.

**Figure 10:**
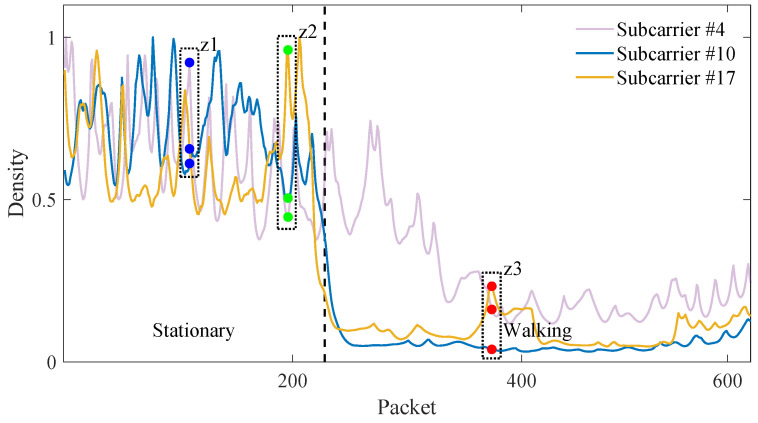
Normalized local density estimation under spatiotemporal dual constraints.

Therefore, we compute the normalized density series and present them in Figure 10. It is evident that although subcarrier #10 exhibits the best overall performance among the three, the instantaneous performance across subcarriers varies significantly over time. Taking the three highlighted points as an example, for points $z_{1}$ and $z_{2}$ located in the static interval, where higher density is desired, subcarriers #4 and #17 perform better. For point $z_{3}$ located in the dynamic interval, where lower density is expected, subcarrier #10 is superior.

**Figure 11:**
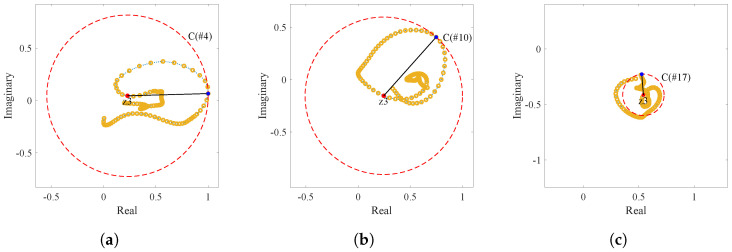
CSI Ratio samples around $z_{3}$ on the complex plane. (**a**) Subcarrier #4. (**b**) Subcarrier #10. (**c**) Subcarrier #17.

The above issue stems from two main factors. First, at a given time instant, the inherent differences in the original CSI Ratio distributions across subcarriers lead to variations in the calculated density values. As Figure 11 shows, the K-distance neighborhood area for subcarriers #4 and #10 are similar and are both much larger than that for subcarrier #17. Consequently, subcarriers #4 and #10 yield significantly lower calculated density values than subcarrier #17. Second, differences in normalization coefficients also alter the normalized density values. For example, the maximum density value of subcarrier #10 is significantly larger than that of subcarrier #4. Thus, although the raw density values for the two subcarriers are comparable, the much larger normalization coefficient for subcarrier #10 results in a smaller normalized value.

The above analysis indicates that the subcarrier-level selection mechanism suffers from a notable problem of instantaneous deviations. To address this, we propose a packet-level asymmetric truncation-based subcarrier fusion method to aggregate information from multiple subcarriers at a finer granularity. Specifically, for each CSI Ratio sample point $z_{i}$, we calculate the improved KNN density values for all $N_{carrier}$ subcarriers, forming a density vector

(11) $$\mathrm{Den}\left(z_{i}\right)=\left[den\left(z_{i},\#1\right),den\left(z_{i},\#2\right),\ldots ,den\left(z_{i},\#N_{carrier}\right)\right]$$

where $den(z_{i},\#j)$ denotes the local density at $z_{i}$ using the $j_{th}$ subcarrier. The elements in $\mathrm{Den}(z_{i})$ are then sorted, and the smallest $N_{low}$ and largest $N_{top}$ values are truncated, which mitigates the influence of outlier subcarriers with poor static stability and weak dynamic sensitivity. The $N_{reserved}$ retained density values across all time instances are consolidated into a two-dimensional density matrix, where $N_{reserved}=\left(N_{carrier}-N_{low}-N_{top}\right)$. This matrix is then used for subsequent motion segmentation. Exhaustive experimental analysis indicates that, compared to dynamic sensitivity, the static stability of subcarriers plays a more crucial role in segmentation accuracy (see Section 5.5.2 for detailed results). Thus, the truncation is configured asymmetrically with $N_{low}>N_{top}$, thereby removing more outlier subcarriers with poor static stability.

**Figure 12:**
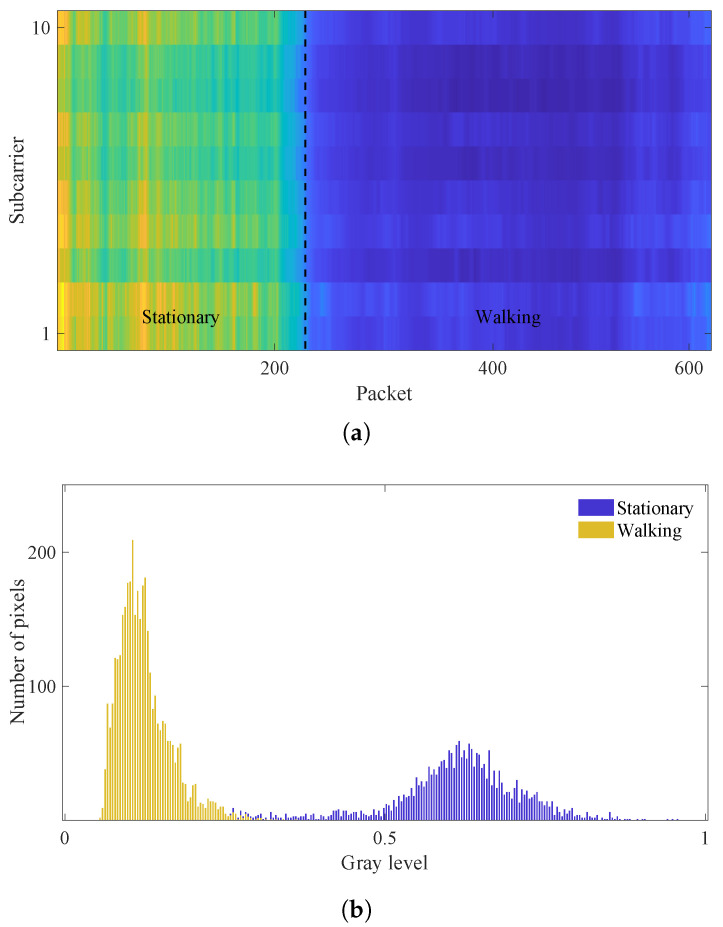
Results of asymmetric truncated subcarrier fusion. (**a**) Image. (**b**) Histogram.

Figure 12a shows the feature representation after asymmetric truncation fusion processing with parameters $N_{carrier}=30$, $N_{low}=15$, and $N_{top}=5$. It can be seen that the packet-level dynamic selection provides an adaptive strategy that enables the real-time selection of optimal subcarriers according to local channel conditions. As a result, both static and motion intervals exhibit high intra-cluster compactness, and the boundary between the two intervals is sharp. Figure 12b further shows the histogram of the truncated density image. Compared with the variance distribution shown in Figure 1b, the density values corresponding to static and dynamic states are distributed in two well-separated intervals, and the overall histogram exhibits a distinct bimodal distribution. This characteristic provides a crucial foundation for subsequent training-free motion interval segmentation.

### 4.6. Motion Segmentation Using VE-Otsu Method

**Figure 13:**
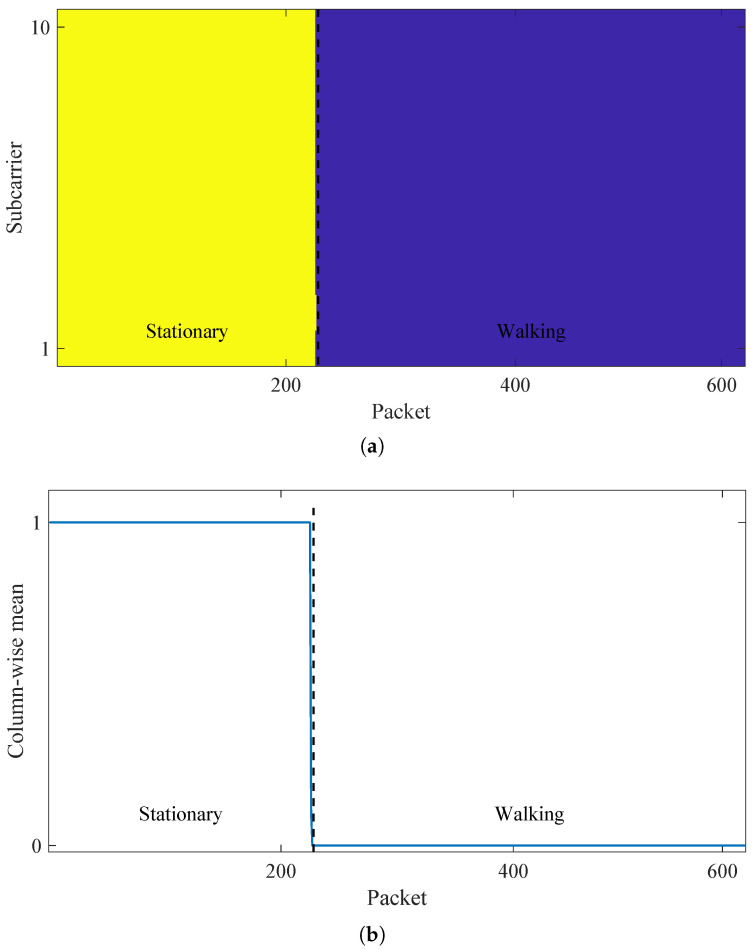
Segmentation result using VE-Otsu method. (**a**) Segmented image. (**b**) Column-wise mean.

To mitigate the potential impact of class imbalance, we employ the VE-Otsu algorithm [43] to segment the motion interval. Then, a morphological operation is applied to the binary result to remove small holes and smooth region boundaries. As shown in Figure 13a, the distinct bimodal distribution of the constructed feature representation enables accurate and complete motion interval segmentation in a single pass. Finally, the motion start and end points are determined by computing the column-wise mean of the binary result as shown in Figure 13b.

## 5. Performance Evaluation

### 5.1. Experiment Setup

**Figure 14:**
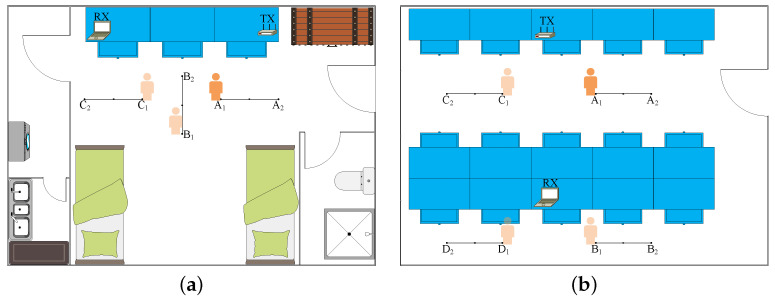
Floor plan of experiment scenarios. (**a**) Dormitory. (**b**) Laboratory.

To evaluate the effectiveness of the proposed method, we conducted comprehensive experiments in two typical indoor environments: a dormitory and a laboratory. As shown in Figure 14, a TP-Link WDR5600 router (TP-Link Co., Ltd., Shenzhen, China) served as the transmitter (TX), and a Lenovo X200 laptop (Lenovo Group Ltd., Beijing, China) equipped with the open-source CSITool [44] acted as the receiver (RX). The sampling rate was set to 100 packets per second.

In the dormitory, five volunteers performed five in-place actions (stepping, squatting, waving, kicking, and sitting down) at three marked positions ($A_{1}$, $B_{1}$, and $C_{1}$). Additionally, each volunteer also performed walking actions, starting from these positions and following the trajectory shown in Figure 14a. In the laboratory, walking actions were conducted from four starting points ($A_{1}$, $B_{1}$, $C_{1}$ and $D_{1}$) as shown in Figure 14b. To ensure consistency, detailed instructions were provided beforehand, and audio prompts were used during collection to guide action timing. The CSI data is then processed by Matlab R2024b (MathWorks, Natick, MA, USA).

### 5.2. Evaluation Metrics

Motion segmentation performance is evaluated in terms of the motion event detection accuracy and the motion interval localization precision. Specifically, a correctly segmented motion interval is defined based on the Temporal Intersection over Union (T-IoU):

(12) $$T-\mathrm{IoU}\left(G,S\right)=\frac{\left|G\cap S\right|}{\left|G\cup S\right|}$$

where *G* and *S* are the ground-truth motion interval and the segmented interval, respectively, and the |G∩S| and |G∪S| denote the duration of the intersection and union of the two time intervals, respectively. A segmented motion interval is considered a True Motion Interval (TMI) if its T−IoU with a ground-truth motion interval exceeds 0.5. Otherwise, it is regarded as a False Motion Interval (FMI).

Furthermore, we employ the Event-level False Positive Rate (E−FPR) and Event-level False Negative Rate (E−FNR) to quantify the motion event detection accuracy of the system, defined as

(13) $$\begin{array}{cc} E-FPR & =\frac{N_{FMI}}{N_{TMI}+N_{FMI}}\\ E-FNR & =\frac{N_{MMI}}{N_{TMI}+N_{MMI}} \end{array}$$

where $N_{TMI}$ and $N_{FMI}$ denote the number of TMI and FMI, respectively, and $N_{MMI}$ is the number of Missed Motion Intervals (MMIs), i.e., those motion events that were not correctly segmented.

To quantify the temporal localization precision of segmented result, we introduce two metrics: the Mean Absolute Error for motion Start point (MAE-S) and the Mean Absolute Error for motion End point (MAE-E). MAE-S is defined as the average of the absolute differences between the segmented motion start times and the ground truth annotations, while MAE-E is defined as the average of the absolute differences between the segmented motion end times and the ground truth annotations. Formally, they are defined as

(14) $$\begin{array}{cc} MAE-S & =\frac{1}{N_{csi}}\sum_{i=1}^{N_{csi}}\left|T_{start,i}^{\left(s\right)}-T_{start,i}^{\left(g\right)}\right|\\ MAE-E & =\frac{1}{N_{csi}}\sum_{i=1}^{N_{csi}}\left|T_{end,i}^{\left(s\right)}-T_{end,i}^{\left(g\right)}\right| \end{array}$$

where $N_{csi}$ is the total number of the collected CSI data, $T_{start,i}^{\left(g\right)}$ and $T_{start,i}^{\left(s\right)}$ are the ground-truth start time and the segmented start time for the $i_{th}$ CSI data, and $T_{end,i}^{\left(g\right)}$ and $T_{end,i}^{\left(s\right)}$ are the ground-truth end time and the segmented end time for the $i_{th}$ CSI data, respectively.

In addition, we evaluate the real-time performance of the algorithm by calculating the average runtime (RT) required to process a single CSI data sample.

### 5.3. Overall Performance

To evaluate the performance of the proposed method, we conducted comparative experiments with several representative approaches, including variance-based LightSeg [17], correlation-based WiSH [21] and SrcSense [23], deep learning-based DeepSeg [29] and LiteWiSys [31], and an image-perspective baseline using variance-envelope-based features [12]. The results are summarized in Table 1. For the learning-based methods, the reported runtime encompasses only the inference phase, excluding the time cost associated with model training.

Regarding motion event detection accuracy, LightSeg, WiSH, and SrcSense rely on empirically set thresholds from static data, rendering them sensitive to noise fluctuations and yielding suboptimal performance. However, owing to its computational simplicity, LightSeg achieves the shortest runtime among non-learning methods. Our method matches the accuracy of the envelope-based approach but with lower runtime. Moreover, our method achieves the lowest motion interval localization error among non-learning methods. This advantage stems from the clear separation and pronounced bimodal distribution of the spatiotemporally constrained density features after asymmetric truncation fusion. The single-pass segmentation of complete motion intervals avoids errors introduced by iterative segmentation and fragment merging, thereby improving localization precision. While DeepSeg and LiteWiSys deliver superior accuracy—demonstrating the power of learned representations—this comes at the high cost of data collection and annotation. In contrast, our training-free method determines its threshold directly from the statistical properties of the density representation, achieving a favorable balance between accuracy and deployment overhead.

Furthermore, although the experimental data covers different scenarios, targets, locations, and activity types, the system shows consistent performance.

### 5.4. Ablation Study

In this subsection, a series of ablation studies are conducted to evaluate the contributions of the spatiotemporally constrained local density estimator and the asymmetric truncation-based subcarrier fusion.

#### 5.4.1. Density Calculation with Spatial-Only Constraints

**Figure 15:**
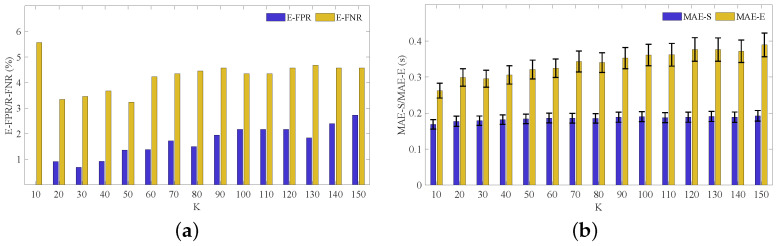
Motion segmentation results using spatial-constraints-only density. (**a**) E-FPR and E-FNR. (**b**) MAE-S and MAE-E.

In this ablation study, we removed the temporal constraints by computing the KNN density based solely on the spatial distribution of CSI Ratio samples on the complex plane. Specifically, we set the neighborhood size *K* and applied Equation (9) to calculate the density, while keeping all other processing steps identical to the full method. Figure 15 shows the resulting system performance under spatial-only constraints for different *K* values.

Density estimation with spatial-only constraints leads to marked decline in both event detection and interval localization accuracy. Even at the optimal K=30, the E-FPR rises to 0.69% and the E-FNR increases sharply to 3.45%, while the mean endpoint localization error increased to 0.30 s. This degradation stems from CSI Ratio trajectory disorder caused by complex human motion. Without the temporal constraints, severely aberrant KNN density values emerge in motion intervals, even overlapping with the static distribution. Even with subcarrier selection, there may still be threshold drift and segmentation fragmentation, thereby degrading the accuracy of both motion event detection and interval localization.

#### 5.4.2. Density Calculation with Temporal-Only Constraints

In a complementary ablation study, we evaluated system performance using a density estimation that considers only temporal constraints. For a sample point $z_{i}$, we consider its two temporal neighbors at a packet-index difference of $N_{index}$, denoted as $z_{i-N_{index}}$ and $z_{i+N_{index}}$. The distance metric is defined as the maximum Euclidean distance to these two points, i.e., $d_{N_{index}}\left(z_{i}\right)=max\left(d\left(z_{i},z_{i-N_{index}}\right),d\left(z_{i},z_{i+N_{index}}\right)\right)$. The local density under temporal-only constraints is then calculated as

(15) $${den}_{T-only}\left(z_{i}\right)=\frac{N_{index}}{N\pi d_{N_{index}}^{2}\left(z_{i}\right)}$$

**Figure 16:**
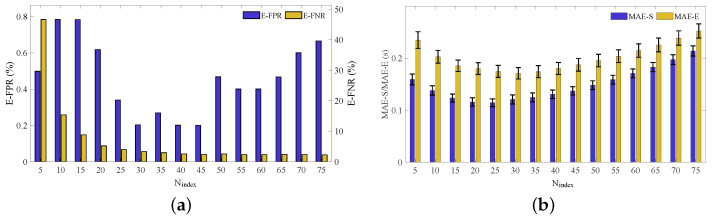
Motion segmentation results using temporal-constraints-only density. (**a**) E-FPR and E-FNR. (**b**) MAE-S and MAE-E.

Figure 16 shows the system performance for different values of $N_{index}$ under this temporal-only configuration. A double y-axis is used for clarity, with the left axis for E-FPR and the right for E-FNR, due to their different value ranges. It can be seen that the system performance under temporal-only constraints degrades substantially in motion event detection accuracy. Even with the optimal parameter $N_{index}=45$, the E-FPR and E-FNR reach 0.20% and 2.40%, respectively, which are considerably higher than those of the full spatiotemporally constrained method. Due to trajectory distortion caused by human motion complexity, the points $z_{i-N_{index}}$ and $z_{i+N_{index}}$ may be abnormally close to $z_{i}$, which yields an excessively small distance metric $d_{N_{index}}\left(z_{i}\right)$ and leads to a severely overestimated local density value. Notably, the optimal $N_{index}$ under temporal-only constraints is smaller than that in the full method. This leads to a sharper boundary between static and dynamic regions and a slight reduction in MAE-S and MAE-E.

Thus, relying solely on spatial or temporal constraints is individually inadequate. The proposed dual spatiotemporal constraints address their respective limitations through a complementary mechanism, which is essential for building a discriminative feature representation.

#### 5.4.3. Effectiveness of Subcarrier Fusion Module

**Figure 17:**
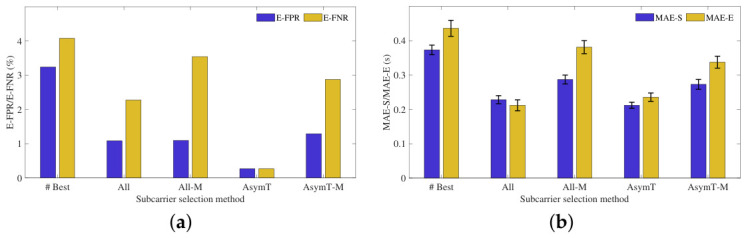
Motion segmentation results using different subcarriers. (**a**) E-FPR and E-FNR. (**b**) MAE-S and MAE-E.

To evaluate the proposed asymmetric truncation fusion algorithm, we systematically compared segmentation performance under different subcarrier selection strategies, with all other processing steps kept identical. The tested strategies were: (1) using each subcarrier individually and selecting the best-performing one (# Best) as the single-subcarrier upper bound; (2) using all subcarriers (All); (3) using the mean series across all subcarriers (All-M); (4) using the proposed asymmetric truncation-based (AsymT) fusion; and (5) using the mean series after asymmetric truncation (AsymT-M). The comparative results are presented in Figure 17.

The results show that relying on a single fixed subcarrier is highly unstable, yielding an E-FPR and E-FNR as high as 3.24% and 4.07%, respectively. Even the best individual subcarrier fails to guarantee consistent performance, as its static stability and dynamic sensitivity fluctuate over time and environment, underscoring the necessity of subcarrier fusion. Adopting an image-based approach using all subcarriers reduces the E-FPR and E-FNR to 1.08% and 2.27%, respectively. This improvement stems from leveraging inter-subcarrier complementarity and incorporating morphological post-processing, which alleviates some missed segmentation issues of single-subcarrier approaches. Furthermore, averaging across subcarriers severely degrades system performance because it averages out the discriminative information present along the subcarrier dimension. Collapsing the 2D feature image into a 1D series not only reduces the sophisticated morphological post-processing to simple time thresholding but also diminishes the bimodality of the histogram due to the drastic reduction in data points. This ultimately introduces errors in threshold calculation and leads to performance decline.

In summary, the proposed packet-level asymmetric truncation achieves dynamic, fine-grained subcarrier selection, overcoming the limitations of both fixed-subcarrier unreliability and the information loss inherent in naive averaging.

### 5.5. Parameters Analysis

#### 5.5.1. Impact of $N_{index}$ and *K*

**Figure 18:**
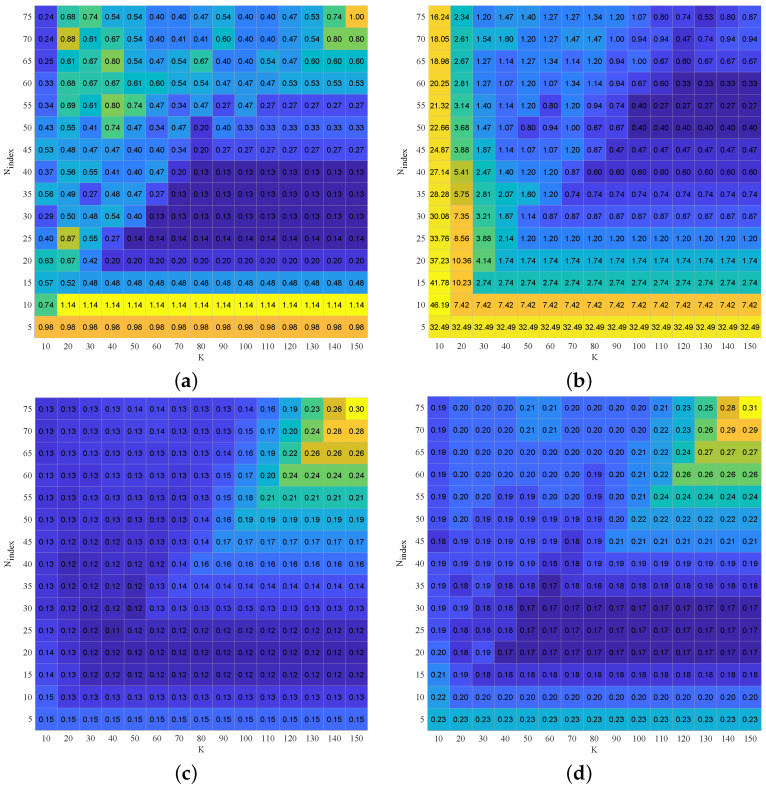
Motion segmentation results using different $N_{index}$ and *K*. (**a**) E-FPR. (**b**) E-FNR. (**c**) MAE-S. (**d**) MAE-E.

The spatiotemporally constrained local density estimation involves two key hyperparameters: $N_{index}$, which governs the temporal correlation range between CSI data points, and *K*, which determines the number of nearest neighbors on the complex plane. To explore their joint impact on segmentation performance, we conducted a systematic grid search over a range of values for both parameters. The results are presented in Figure 18.

Since the $K_{th}$ nearest neighbor is selected from the temporal subset $D_{index}$, the constraint $K\leq 2N_{index}$ must hold. Thus, only the upper triangular region in Figure 18 (where $K\leq 2N_{index}$) represents valid configurations. For completeness, the invalid region ($K>2N_{index}$) is filled with the performance obtained at $K=2N_{index}$. Overall, motion event detection accuracy declines sharply and then increases gradually as $N_{index}$ increases. For a fixed $N_{index}\leq 65$, both E-FPR and E-FNR decrease with *K*. However, for $N_{index}>65$, they first drop sharply and then rise slowly as *K* increases. Specifically, when $N_{index}$ is too small (e.g., 5), the temporal subset $D_{index}$ contains too few samples. This results in underestimated density for motion intervals and poor distinction from static states regardless of the value of *K*, leading to severe threshold misestimation and high E-FPR and E-FNR. With a moderate $N_{index}$ (e.g., 55), $D_{index}$ contains sufficient samples. Adjusting *K* can then produce density features with better discrimination, achieving optimal performance. However, when $N_{index}$ is excessively large (e.g., 75), performance initially improves as *K* increases up to 130. Beyond this point, an overly large *K* results in an extended neighbor search radius within static intervals. This reduces the density contrast, leading to a moderate increase in motion event detection errors.

Regarding motion interval localization accuracy, both MAE-S and MAE-E increase with larger values of $N_{index}$ and *K*. The reason is that these parameters co-determine the temporal neighborhood for density calculation. Larger values yield broader temporal coverage, which smooths the local density series around the boundary between static and dynamic states. This results in a wider transition zone, increasing boundary localization uncertainty and thereby elevating both MAE-S and MAE-E. Additionally, MAE-E was consistently higher than the MAE-S. This difference stems from the nature of human activity: movement initiation is typically abrupt, whereas stopping involves a gradual deceleration. This inherent asymmetry makes the transition at motion end more ambiguous in the density series, thus resulting in a slightly higher MAE-E.

Furthermore, the experimental results show that a wide range of $N_{index}$ and *K* values yield satisfactory performance across all four metrics. Prioritizing motion event count accuracy, the combination $N_{index}=55$ and K=110 was therefore chosen and applied in all subsequent experiments.

#### 5.5.2. Impact of $N_{low}$ and $N_{top}$

**Figure 19:**
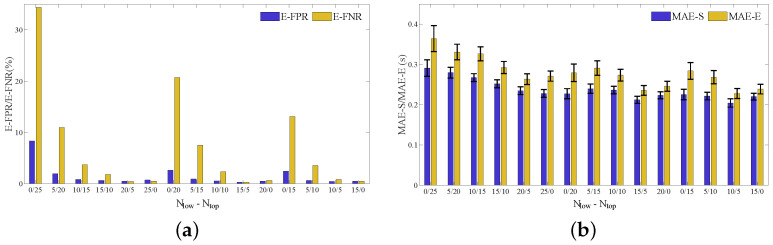
Motion segmentation results using different $N_{low}$ and $N_{top}$. (**a**) E-FPR and E-FNR. (**b**) MAE-S and MAE-E.

In the asymmetric truncated subcarrier fusion, the parameters $N_{low}$ and $N_{top}$ determine the number of local density minima and maxima truncated for each time instance, respectively. This process aims to select subcarriers with strong static stability and dynamic sensitivity. To investigate their impact on segmentation, we tested the system performance under different combinations of $N_{low}$ and $N_{top}$. The experimental results are shown in Figure 19, where the horizontal axis is labeled in the format $N_{low}/N_{top}$, indicating the truncation of $N_{low}$ minima and $N_{top}$ maxima per time instance.

The effectiveness of asymmetric truncation-based fusion method critically depends on co-optimizing the number of reserved subcarriers and the truncation position. First, the total number of subcarriers retained per instance $N_{reserved}$ must lie within a reasonable range. First, the total number of subcarriers retained per instance $N_{reserved}$ must lie within a reasonable range. Experiments identify an optimal value of 10 for $N_{reserved}$, which yields better performance than those of 5 and 15. If $N_{reserved}$ is too small (e.g., 5), it leads to over-truncation. This excessively compresses information along the subcarrier dimension and leaves insufficient features for robust thresholding. Conversely, if $N_{reserved}$ is too large (e.g., 15), under-truncation occurs, which fails to adequately filter out outlier subcarriers with poor static stability or dynamic sensitivity. Both scenarios weaken inter-class feature discriminability and increase segmentation difficulty.

Compared with the quantity of reserved subcarriers, the truncation position is more critical. For a fixed retention of 10 subcarriers, performance is poor when only maxima are truncated at each time instance ($N_{low}=0,N_{top}=20,E-FPR=2.63\%$, and E−FNR=20.72%). This is because subcarriers with poor static stability cause density overlap between static and dynamic states, severely disrupting threshold calculation. In contrast, truncating five subcarriers with the worst static stability markedly improves segmentation accuracy ($N_{low}=5,N_{top}=15$, E−FPR=0.93%, and E−FNR=7.48%), confirming that static stability is the dominant factor. Meanwhile, dynamic sensitivity has a moderating effect. For example, with $N_{low}=15$ and $N_{top}=5$, the system marginally outperforms the configuration $N_{low}=20$ and $N_{top}=0$, which indicates that removing a few outliers with poor dynamic sensitivity promotes tighter intra-class clustering in motion intervals, thereby refining the initial segmentation.

In this study, we set $N_{low}=15$ and $N_{top}=5$ as the optimal parameters. This configuration strikes a balance between retaining sufficient information and effectively suppressing poor subcarriers, thereby maximizing the discriminative power of the fused features and establishing a solid foundation for robust motion segmentation.

### 5.6. Discussion

**Figure 20:**
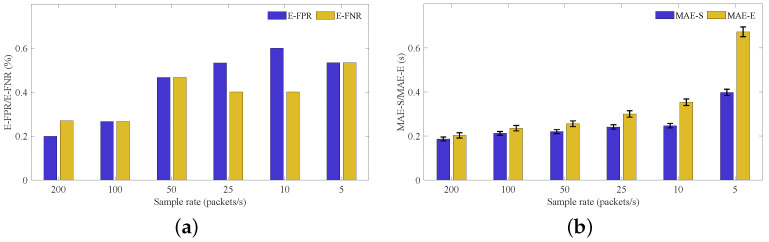
Motion segmentation results with different sampling rate. (**a**) E-FPR and E-FNR. (**b**) MAE-S and MAE-E.

**Sampling rata.** To evaluate the performance of the proposed method under different sampling rates, we down-sampled the original CSI series. The hyperparameters $N_{index}$ and *K* were scaled proportionally. As Figure 20 shows, the motion event detection accuracy remains robust across different sampling rates. Even at 5 packets/s, the E-FPR and E-FNR remain below 0.6%. This robustness is due to the rate-invariant characteristic of the CSI Ratio’s distribution on the complex plane. With appropriate scaling of the temporal parameters, the improved density features can still be extracted even at low rates, ensuring stable motion event detection accuracy. On the other hand, the reduced sampling rate degrades temporal resolution, which adversely affects interval localization accuracy. For example, at 5 packets/s, the MAE-S and MAE-E increase to 0.40 s and 0.67 s, respectively, significantly higher than the 0.21 s and 0.24 s achieved at 100 packets/s. However, an excessively high sample rate provides diminishing improvement. For example, when the sample rate is increased to 200 packets/s via interpolation, only the E-FPR shows a marginal reduction (from 0.27% to 0.21%), while the other three metrics remain virtually unchanged. This negligible gain is outweighed by the doubled computational load, which compromises real-time feasibility. Hence, the selection of sampling rate involves a trade-off between temporal localization accuracy and computational cost. A higher rate is required for precise boundary estimation, whereas a lower rate can significantly improve processing efficiency if only motion event detection is needed.

**Figure 21:**
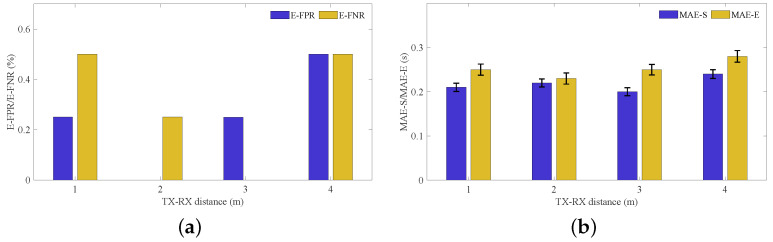
Motion segmentation results with different TX-RX distance. (**a**) E-FPR and E-FNR. (**b**) MAE-S and MAE-E.

**TX-RX distance.** To evaluate the impact of transceiver distance, we tested motion segmentation performance with the transmitter and receiver placed 1, 2, 3, and 4 m apart. The results are shown in Figure 21. Although system performance remains stable across a range of distances, both excessively short and long separations degrade segmentation accuracy. For instance, performance at 1 m and 4 m is slightly worse than that at 2 m and 3 m. When the distance is too short, the strong LOS component diminishes the relative contribution of the dynamic component induced by human motion. Conversely, an excessively long distance increases the propagation path length and signal attenuation of the dynamic component. Both scenarios are detrimental to accurate motion interval segmentation.

**NLOS condition.** We further evaluated the proposed method under Non-Line-of-Sight (NLOS) conditions. Specifically, with the transmitter stationary, the receiver was placed inside the bathroom, with a wall blocking the direct path. Table 2 presents the motion segmentation results under NLOS. The results show a moderate degradation in system performance. This is because, in NLOS scenarios, the wireless signal reflected by the moving human body undergoes multiple reflections and diffractions before reaching the receiver. This process significantly attenuates the dynamic component, thereby reducing the channel perturbation caused by human motion. Consequently, the distinction between static and dynamic states is diminished, increasing the segmentation difficulty.

**Unseen environment.** To verify the generalization capability, we evaluated the proposed method on a new public dataset [45]. As shown in Table 2, despite differences in room setup, hardware, subjects, and activity types compared to our self-collected dataset, the proposed method still achieves accurate motion interval segmentation. This validates the effectiveness of the spatiotemporally constrained density estimator and the packet-level asymmetric subcarrier fusion. For comparison, we also tested DeepSeg and LiteWiSys in this new dataset, and found that the models trained on the original data showed degraded performance, with E-FPRs of 2.37 and 1.02, and E-FNRs of 1.25 and 0.97, respectively. However, after fine-tuning with a small amount of new data, both methods showed substantial improvement, with E-FPRs dropping to 0.22 and 0.26, and E-FNRs to 0.28 and 0.20, respectively. In contrast, the proposed method requires no data collection or model training for new scenarios, demonstrating an advantage in terms of lower deployment overhead.

**Noisy environment.** We introduced an operating fan and an intermittently moving robot to simulate real-world environmental disturbances. The system performance under these two types of interference is shown in Table 2. The results indicate that other moving objects significantly degrade segmentation performance, especially under the strong interference from the robot. This is primarily due to the limited multi-target resolution capability imposed by the narrow Wi-Fi signal bandwidth. This limitation makes it difficult for the improved KNN density estimator to distinguish whether changes in the spatial distribution of the CSI Ratio originate from genuine human motion or environmental interference. In the future, exploring more effective filtering methods in data preprocessing [46] or leveraging powerful deep learning models may provide new directions for robust motion segmentation under strong interference.

## 6. Conclusions

Human motion interval detection and segmentation is a core task for device-free Wi-Fi sensing. This paper introduces a training-free method that integrates spatiotemporally constrained density estimation with packet-level asymmetric subcarrier fusion for discriminative feature representation. Real-world experiments validate its accuracy in both motion event detection and interval localization. Future work will focus on motion segmentation in more complex environments and extending the framework to advanced sensing tasks.

## Figures and Tables

**Table 1:** System performance compared with other methods.

Method	E-FPR (%)	E-FNR (%)	MAE-S (s)	MAE-E (s)	RT (s)
LightSeg	0.89	1.34	0.48	0.57	0.17
WiSH	0.78	1.00	0.27	0.29	0.38
SrcSense	0.81	0.54	0.35	0.38	0.41
DeepSeg	0.21	0.25	0.32	0.38	0.06
LiteWiSys	0.27	0.16	0.19	0.21	0.04
Envelope-based	0.33	0.20	0.26	0.29	0.26
Ours	0.27	0.27	0.21	0.24	0.23

**Table 2:** System performance in complex conditions.

Conditions	E-FPR (%)	E-FNR (%)	MAE-S (s)	MAE-E (s)
NLOS	1.77	2.75	0.28	0.34
Human to human	0.30	0.28	0.23	0.26
Fan interference	2.02	3.00	0.38	0.51
Robot interference	14.29	28.00	0.68	0.89

## Data Availability

The data presented in this study are available on request from the corresponding author. The data are not publicly available due to privacy reasons.

## Abbreviations

The following abbreviations are used in this manuscript:

RSSI	Received Signal Strength Indicator
CSI	Channel State Information
SVM	Support Vector Machine
ACF	Autocorrelation Function
CNN	Convolutional Neural Network
LSTM	Long Short-Term Memory
FFT	Fast Fourier Transform
CFAR	Constant False Alarm Rate
STFT	Short Time Fourier Transform
BNR	Breath-to-Noise Ratio
MIMO	Multiple Input Multiple Output
OFDM	Orthogonal Frequency Division Multiplexing
LOS	Line of Sight
AGC	Auto Gain Control
CFO	Carrier Frequency Offset
SFO	Sampling Frequency Offset
STO	Symbol Timing Offset
SNR	Signal-to-Noise Ratio
KNN	K-Nearest Neighbor
PDF	Probability Density Function
VE-Otsu	Valley-Emphasized Otsu
T-IoU	Temporal Intersection over Union
E-FPR	Event-level False Positive Rate
E-FNR	Event-level False Negative Rate
MAE-S	Mean Absolute Error for motion Start point
MAE-E	Mean Absolute Error for motion End point
